# Initial Assessment of Variability of Responses to Toxicants in Donor-Specific Endothelial Colony Forming Cells

**DOI:** 10.3389/fpubh.2018.00369

**Published:** 2018-12-21

**Authors:** Daria Filonov, Raymond Tice, Ruiyan Luo, Chad Grotegut, Michael J. Van Kanegan, John W. Ludlow, Dora Il'yasova, Alexander Kinev

**Affiliations:** ^1^Creative Scientist, Inc. Durham, NC, United States; ^2^School of Public Health, Georgia State University, Atlanta, GA, United States; ^3^Duke University Medical Center, Durham, NC, United States; ^4^Zen-Bio, Inc, Research Triangle Park, NC, United States

**Keywords:** toxicological risk assessment, animal replacement, population variability, endothelial cells, developmental toxicants, cardiovascular disease, personalized medicine

## Abstract

There is increased interest in using high throughput *in vitro* assays to characterize human population variability in response to toxicants and drugs. Utilizing primary human endothelial colony-forming cells (ECFCs) isolated from blood would be highly useful for this purpose because these cells are involved in neonatal and adult vasculogenesis. We characterized the cytotoxicity of four known toxic chemicals (NaAsO_2_, CdCl_2_, tributyltin [TBT], and menadione) and their four relatively nontoxic counterparts (Na_2_HAsO_4_, ZnCl_2_, SnCl_2_, and phytonadione, respectively) in eight ECFC clones representing four neonatal donors (2 male and 2 female donors, 2 clones per donor). ECFCs were exposed to 9 concentrations of each chemical in duplicate; cell viability was evaluated 48 h later using the fluorescent vital dye fluorescent dye 5-Carboxyfluorescein Diacetate (CFDA), yielding concentration-effect curves from each experiment. Technical (day-to-day) variability of the assay, assessed from three independent experiments, was low: *p*-values for the differences of results were 0.74 and 0.64 for the comparison of day 2 vs. day 1 and day 3 vs. day 1, respectively. The statistical analysis used to compare the entire concentration-effect curves has revealed significant differences in levels of cytotoxicity induced by the toxic and relatively nontoxic chemical counterparts, demonstrating that donor-specific ECFCs can clearly differentiate between these two groups of chemicals. Partitioning of the total variance in the nested design assessed the contributions of between-clone and between-donor variability for different levels of cytotoxicity. Individual ECFC clones demonstrated highly reproducible responses to the chemicals. The most toxic chemical was TBT, followed by NaAsO_2_, CdCl_2_, and Menadione. Nontoxic counterparts exhibited low cytotoxicity at the higher end of concentration ranges tested. Low variability was observed between ECFC clones obtained from the same donor or different donors for CdCl_2_, NaAsO_2_, and TBT, but for menadione, the between-donor variability was much greater than the between-clone variability. The low between-clone variability indicates that an ECFC clone may represent an individual donor in cell-based assays, although this finding must be confirmed using a larger number of donors. Such confirmation would demonstrate that an *in vitro* ECFC-based testing platform can be used to characterize the inter-individual variability of neonatal ECFCs exposed to drugs and/or environmental toxicants.

## Introduction

The response of individual humans to hazardous exposures can vary significantly and this variability is thought to underlie individual predispositions to to diseases and/or sensitivity to toxic exposures (1). While basic biological mechanisms pertinent to humans are being studied in laboratory animals, the variability of human responses to chemicals, including drugs (2) and environmental toxicants (3), cannot be adequately assessed by traditional animal models. This limitation was the primary reason for the development of two new mouse model systems—the Collaborative Cross and the Diversity Outbred (4–7)—that could be used to study how genetic polymorphisms alter sensitivity to the adverse effects of chemicals. However, data generated using these animal models still requires extrapolation to human populations and is unlikely to substitute for all human variability.

This limitation has increased scientific interest in using high throughput (HT) *in vitro* cell-based systems to evaluate the extent of human genetic and functional variability in response to chemical toxicants. In 2012, Lock et al. (8) reported on the differential sensitivity of 81 human lymphoblastoid cell lines from 27 Center d'Etude du Polymorphisme Humain trios exposed to 240 chemicals using cytotoxicity and apoptosis as endpoints in a quantitative HT screening platform. These investigators concluded that an evaluation of toxicity pathways and the effects of genetic diversity was now feasible. Subsequently, in 2015, Abdo et al. (9) expanded this approach by testing the variability in cytotoxicity to 179 chemicals using lymphoblastoid cell lines representing 1,083 individuals from European, Chinese, Japanese, African, and Hispanic ancestries. The difference in donor-specific cellular responses measured as an EC10 (effective concentration by which control culture cell viability was reduced by 10%) for about half of the tested compounds was found to vary between 10- and 1,000-fold (9). These data were used to develop prediction models for human population responses to toxic chemicals (10), further indicating the value of the approach.

The large difference in donor-specific cellular reactions identified for some chemicals by Abdo et al. (9) provides unequivocal evidence that human individual variability in response to toxicants can be studied in cell-based models and should be carefully considered in population-wide assessments of toxicological risks. Both Lock et al. (8) and Abdo et al. (9) used human lymphoblastoid cell lines because those were available well-defined cells that would allow for a population characterization. However, recent advance in the isolation and characterization of human stem and progenitor cells and in the generation of induced pluripotent stem cells (iPSCs) suggests that populations of normal rather than transformed (i.e., lymphoblastoid) cells could be used for the same purpose. Moreover, the lineage-committed progenitor cells might be particularly useful for evaluating the variability of human responses to toxicants in specific types of human tissues or organs and/or processes where these cells play key roles.

We previously suggested that utilizing progenitor cells isolated from human umbilical cord fits the described framework of population-based toxicological testing (11). Formed during fetal development, these progenitor cells can be harvested from the umbilical cord at birth, which provides a non-invasive procedure for establishing a population-based collection of cells whose previous exposure to the environment is limited to *in utero* conditions. Accordingly, the collected cells would exhibit a minimum of acquired non- or epi-genetic modifications that might potentially affect their responses to chemicals beyond the inherent genetics. Specifically, cord blood-derived endothelial progenitor cells could serve as a model for a population-based platform for screening environmental toxicants with a potential for exerting vascular toxicity (11). This information may be relevant to individual developmental and cardiovascular risks arising from functional deficits as a result of exposures to toxicants. *In vivo* endothelial progenitor cells are involved in blood vessels formation during both *in utero* development and postnatally (12–15) and the vasculature is the first and largest organ in the developing embryo/fetus (16, 17). The existence of functioning (healthy) vessels is a prerequisite for proper development and function of all other tissues and organs. Therefore, endothelial toxicity has a clear potential to affect the developmental path of many organs and tissues (18, 19). In this study, we present the first step in building a platform for screening of drugs and environmental toxicants for endothelial toxicity.

Endothelial colony-forming cells (ECFCs) is a sub-set of endothelial progenitor cells committed to endothelial lineage. A considerable body of work has demonstrated that these cells exhibit vasculogenic properties during periods of high demand for vessel growth, such as embryonic development and ischemia (20). ECFCs received their name because after isolation, a single proliferating endothelial progenitor cell can produce a colony of several thousand descendants which, with sub-culturing, can give rise to millions of cells (21, 22). Under optimal growth conditions, several dozens of ECFC clones can be obtained from each donor. Therefore, to evaluate donor-specificity of *in vitro* ECFC responses to chemicals, we isolated several ECFC clones from each individual cord blood sample. In this study, we took eight ECFC clones from four donor samples (two clones per donor) and measured changes in viability of the ECFC clones in response to toxic compounds. We compared differences between clones derived from the same donor (within-person variability) to differences of responses between donors (between-person variability). We presumed that, if the within-person variability is less than the between-person variability, then the ECFCs responses to toxicants are donor-specific.

We selected four chemicals with demonstrated endothelial cell and/or vascular toxicity and four relatively nontoxic chemicals as counterparts. The chemical pairs tested were: (1) cadmium (Cd, as CdCl_2_) and zinc (Zn, as ZnCl_2_), (2) arsenic (III) (as NaAsO_2_) and arsenic (V) (as Na_2_HAsO_4_), (3) tin-containing compounds tributyltin (TBT) and SnCl_2_, and (4) a toxic vitamin K family member menadione (vitamin K3, or 2-methyl-1,4-naphthoquinone) and nontoxic phytonadione (Vitamin K1, also known as phylloquinone). Cadmium and arsenic enter the human body via contaminated food, drinking water, and tobacco smoke (23). Cadmium and trivalent arsenic, or arsenite, are developmental and cardiovascular toxicants, which are known to impair the function of endothelial cells (24–28). TBT is an environmental contaminant known for its developmental toxicity, particularly in aquatic species, which also exerts endothelial cell toxicity (29–32). Menadione is a synthetic form of vitamin K, which is used as a nutritional and medicinal supplement in animals and sometimes in humans in low income countries (33) despite its well-known ability to stimulate oxidative stress *in vitro* (34–36) and a demonstrated toxicity toward endothelial cells (36, 37). To determine whether each individual ECFC clone can correctly distinguish a more potent chemical from its less potent counterpart, we selected four relatively nontoxic pairs to these toxicants. Specifically, we used ZnCl_2_ as a relevant pair to CdCl_2_ because the potential mechanism of Cd^2+^ toxicity involves competition with Zn^2+^, an essential cofactor in proteins (38–41); sodium arsenate (or pentavalent As, Na_2_HAsO_4_) as a less toxic counterpart to trivalent As (42–44); inorganic tin, SnCl_2_, as a less toxic counterpart of TBT (45, 46); and natural vitamin K1 (phytonadione) as a nontoxic matching pair to menadione (33).

## Materials and Methods

### Isolation of ECFCs

ECFC clones were isolated from cord blood specimens obtained at delivery from 2 girls and 2 boys born to non-smoking mothers recruited to this study with signed consent form at Duke University Medical Center (DUMC, Durham, NC). The Institutional Review Boards of DUMC and Georgia State University approved this research protocol.

The cord blood samples were collected into 4 mL Sodium Heparin BD Vacutainer® tubes (cat. #367871, BD Biosciences). Samples were maintained at room temperature and processed within 12 h after collection. All reagents and buffers were brought to room temperature before sample processing. The blood samples were transferred from vacutainers into sterile 5 mL tubes (cat. #24-285SCS, Genesee Scientific) and 1/10th volume of 100 mg/mL dextran solution (cat. #31392, MilliporeSigma) was mixed with each sample. Tubes were incubated for 15 min at room temperature and then centrifuged in Beckman Coulter Allegra 6R Bench Top centrifuge for 10 min at 250 rpm to pellet red blood cells. The supernatant containing plasma and white blood cells was collected, transferred to a new sterile 5 mL tube, and centrifuged again for 10 min at 1,400 rpm to pellet the remaining cells. After centrifugation, the plasma was removed, and the cell pellet was resuspended in 5 mL Hanks's Balanced Salt Solution with Ca^2+^ and Mg^2+^ (HBSS-Ca^2+^/Mg^2+^, cat. #21-023-CV, Corning, Inc.). Cells were pelleted again by centrifugation for 10 min at 1,200 rpm. This wash step was repeated one more time and after the second wash, the cells were resuspended in 4 mL of Vecstem^TM^ endothelial cell growth media (Creative Scientist, Inc.) and counted in fluorescence mode using the cell counter Luna FL (Logos Biosystems, Inc.). The cells were then equally divided and plated in 2 wells on a 6-well Primaria^TM^ plate (cat. #353846, Corning, Inc.) at 2 mL/well. On each of the following three days, the plates were washed three times with room temperature HBSS without Ca^2+^ and Mg^2+^ (HBSS, cat. #CS-022-CV, Corning, Inc.) and fresh Vecstem^TM^ added. Starting on day three, the plates were examined for the presence of colonies under a microscope at 10x magnification. When colonies became visible, they were collected using sterile glass clonal rings (cat. #C1059, MilliporeSigma). For this, the medium was removed and 1 mL/well of HBSS was added. The clonal ring was carefully placed over the colony; cells were detached by incubation with 50 μL of 0.05% Trypsin/0.53 mM EDTA (cat. #25-051-Cl, Corning, Inc.) and transferred to a new 6-well plate, 1 colony/well. The cells were expanded for 3 passages and frozen for future use.

### Cell Surface Marker Characterization

For cell surface marker expression analysis using flow cytometry, the cells were first expanded in 10 cm dishes in Vecstem^TM^ media. For staining, cells were washed with cold phosphate buffered saline (DPBS, cat. #21-031-CM, Corning, Inc.) and treated for 3 min with accutase solution (cat. #C41310, PromoCell). Detached cells were collected and centrifuged at 300 x g for 5 min, then washed with labeling buffer PBS, pH 7.2 with 0.5% bovine serum albumin (BSA, cat. #A2153, MilliporeSigma) and 2 mM EDTA (cat. #15575020, Thermo Scientific). Two lakhs of cells were incubated with conjugated antibodies (Supplemental Table 1) for 1 h. according to manufacturers' specifications. After incubation, the cells were washed with and resuspended in 100 μL of labeling buffer. Unlabeled cells were used to establish side scatter and fluorescent gating and intensity measurements of >10,000 cells. Data were recorded using MACSQuant Analyzer 10 (Miltenyi) and were exported as FCS files; median fluorescence for each sample was analyzed using FCS Express 6 (De Novo Software).

### Proliferation Assay

ECFCs were seeded in 384 well μClear plates (cat. # 781091, Greiner Bio-One) at 500 cells/well in 40 μL of Vecstem^TM^ (Creative Scientist, Inc.). The plate outline is provided as Supplemental Figure 1. The next day, 10 μL of 1:1 serially diluted toxicants were added to cells in duplicate (2 wells per concentration of each chemical, 9 titers). All chemicals used in the current study are listed in Supplemental Table 2. Selection of the concentration range for each toxic chemical was based on published data and on preliminary experiments (data not provided). The concentration range for the nontoxic chemical counterparts was chosen to overlap the concentration range of the toxic chemical. After 48 h, cell growth was assessed using the vital dye 5-carboxyfluorescein diacetate (CFDA, cat. #C4916, MilliporeSigma). Cell growth media was removed from wells and cells were washed once with 40 μL HBSS-Ca^2+^/Mg^2+^. Subsequently, 10 μL of 1.5 μM CFDA of in HBSS with Ca^2+^ and Mg^2+^ were added per each well. The cells were incubated for 1 hr and fluorescence was read at λ_ex_/λ_em_ 485 nm/525 nm using fluorescent plate reader Infinite F200 (TECAN) using Integration Time−20 μs, Gain−50, and Number of Flashes−50. The experiment was repeated on three separate days (*n* = 3).

### Proliferation Assay Data Normalization and Transformation

The fluorescent intensity for each toxicant concentration on a plate (*n* = 2) was averaged and normalized to the average fluorescent intensity of untreated control wells (*n* = 4). To account for non-specific drift of the baseline sometimes observed in cell-based assays using high-throughput plates, each chemical had two pairs of wells with untreated controls—at the beginning and the end of the chemical titer range as shown in Supplemental Figure 1. Thus, for each clone in each plate there were total 32 wells with untreated controls. Fewer than 10% of the control wells were identified as outliers based on analysis using GraphPad Prizm 7.04 program and were excluded from further analysis. The normalized values of cell viability per each chemical tier from each independent experiment were then averaged and plotted against concentration for each chemical.

### Statistical Analysis

#### Non-linear Fit of Concentration-Response Curves

To estimate differences between individual concentration response curves, we used statistical methodology based on the functional data analysis of concentration-effect curves (47). To begin with, we fitted a concentration-effect curve for each experiment as follows. Let *x*_*j*_, *j* = 1, ⋯ , *J*, be the measured cell viability value at the *j*-th concentration level *t*_*j*_ expressed as log_2_(1+μM). Let *y*(*t*) be the true concentration-effect curve to be fitted. Considering the presence of measurement error, we assume that

$$\begin{array}{l} x_{j}=y\left(t_{j}\right)+\varepsilon_{j} \end{array}$$

where ε_*j*_ is the noise contributing to a roughness to the raw data. It is known that *y*(*t*) is a smooth curve and monotonically decreases from 100 to 0 as *t* increases from 0. We express *y*(*t*) as

$$\begin{array}{l} y\left(t\right)=\frac{100}{1+\int_{0}^{t}e^{\sum_{k=1}^{K}c_{k}b_{k}\left(s\right)}ds} \end{array}$$

where [*b*_1_(*s*), ⋯ , *b*_*K*_(*s*)] are *K* basis functions, and (*c*_1_, ⋯ , *c*_*K*_) are the corresponding expansion coefficients. Basis functions are mathematically independent and can approximate arbitrarily well any function by taking a weighted sum of a sufficient large number *K* of these functions (47). To estimate the expansion coefficients *c*_1_, ⋯ , *c*_*K*_, we adopt the penalized least squares criterion and minimize the penalized sum of squared error

$$\begin{array}{l} \text{PENSSE }\left(y|x\right)=\overset{J}{\underset{j=1}{\sum }}{\left\{x_{j}-y\left(t_{j}\right)\right\}}^{2}+\lambda \times \text{PEN}\left(y\right) \end{array}$$

where $\overset{J}{\underset{j=1}{\sum }}{\left\{x_{j}-y\left(t_{j}\right)\right\}}^{2}$is the sum of squared error, $\text{PEN}\left(y\right)=\int {\left\{\sum_{k=1}^{K}c_{k}b_{k}^{\prime \prime }(t)\right\}}^{2}dt$ is the roughness penalty with ${b_{k}}^{\prime \prime }\left(t\right)$ representing the second derivative of *b*_*k*_(*t*), and λ>0 is a smoothing parameter. Here the roughness of *y*(*t*) is defined as the roughness of $\overset{K}{\underset{k=1}{\sum }}c_{k}b_{k}\left(t\right)$, the logarithm of the derivative of the reciprocal of *y*(*t*). Highly variable functions can be expected to yield high values of PEN(*y*). This penalized least squares criterion trades off smoothness against data fit, and the smoothing parameter λ measures the rate of exchange between fit to the data and variability of the function *y*(*t*). Here we choose *K* = 20 B-spline basis functions (48) and λ = 0.01, and estimate the coefficients *c*_1_, ⋯ , *c*_*K*_ by minimizing PENSSE (*y*|*x*) using the Newton-Raphson method.

#### Assessment of Day-To-Day (i.e., Technical) Variability

Initially, we tested technical variability of our assay by establishing concentration-response curves for four toxic chemicals: CdCl_2_, NaAsO_2_, TBT, and menadione. In these initial experiments, we used a polyclonal ECFC line CB002, which we used in previous work (21). Each experiment was performed as described above in duplicates and repeated three times on separate days (Supplemental Figure 2). Then we fitted the curves for a total of 12 concentration-response curves (four chemicals × 3 days). The curves were used to examine the extent of day-to-day variability of our assay, considering differences in the effects of toxicants. In this analysis, we fit the following functional ANOVA model (47):

(1) $$\begin{array}{r} Y_{ij}\left(t\right)=\mu \left(t\right)+\alpha_{i}\left(t\right)+\beta_{j}\left(t\right)+\varepsilon_{ij}\left(t\right) \end{array}$$

where *Y*_*ij*_(*t*) represents the fitted concentration-effect curve of the i-th toxicant on the j-th day, t–represents the concentration level and is expressed as log_2_(1 + μM). To ensure the capacity of the model to identify different curves, we restrict α_1_(*t*) = β_1_(*t*) = 0. Then μ(*t*) represents the mean concentration-effect curve of the first toxicant on the first day, α_*i*_(*t*) represents the difference of the mean concentration-effect curve of the i-th toxicant compared to the first toxicant on the same day, β_*j*_(*t*) represents the difference of the mean concentration-effect curve of the j-th day compared to the first day for the same toxicant, and ε_*ij*_(*t*) represents the noise curve. This model was fitted using the *fosr* function in the **R** package “refund” (49) and the estimated β_2_(*t*) and β_3_(*t*) comparisons are shown in Supplemental Figure 3.

#### Assessment of Within- and Between-Donor (Biological) Variability

To evaluate the contribution of within- and between-donor variability, we examined responses of eight ECFC lines from four donors (2 clones × 4 donors) to eight chemicals using two replicates of each titer of chemicals in each experiment repeated on three separate days. With the method described above, we fitted a concentration-effect curve using the available values for cellular viability and concentration levels for each clone/each replicate/each toxicant. A total of 22 fitted concentration-effect curves were analyzed for each tested chemical. Using optimized fitted curves, we determined the concentration corresponding to a specific toxic effect for each curve. For example, given a fitted curve ŷ(*t*), to determine an IC10 (the inhibitory concentration that reduces viability compared to the control data by 10%), we first obtained the values of ŷ(*t*) at 300 equally spaced toxicant concentration levels 0 = *t*_1_<*t*_2_ < … < *t*_300_ = *T*, where *T* is a value slightly higher than the maximum observed concentration level [i.e., log_2_(1 + μM)] for the examined chemical. As ŷ(*t*) monotonicly decreases from 100 to 0, we determine *i* such that ŷ(*t*_*i*_) > 10 > ŷ(*t*_*i*+1_). Then we find the value of *IC*10 by the formula

$$\begin{array}{l} \text{IC}10=2^{{\hat{t}}_{10}}-1,\text{ where }{\hat{t}}_{10}=t_{i}-\frac{ŷ\left(t_{i}\right)-10}{ŷ\left(t_{i}\right)-ŷ\left(t_{i+1}\right)}\left(t_{i}-t_{i+1}\right) \end{array}$$

so that $ŷ\left(\;{\hat{t}}_{10}\right)=$10 using linear interpolation. We could not calculate IC10 values for most of the less toxic counterparts of the examined chemical pairs because a 10% loss of viability was not observed. Finally, using the *lm* function in **R** to fit a linear regression model with donor and clone (nested within donor) as covariates, we calculated total, between-donor, between-clone, and within-clone (technical) sum square variability for each examined toxic effect (i.e., for 10, 20, 30%, etc.) and presented the proportion of between-person/between-clone and between-person/total sum square variability for each effect size (see Results).

### Graphical Data Presentation

Data were plotted in GraphPad Prizm 7.04 software.

## Results

### Characterization of Cord-Blood (CB) ECFCs

Typically, we received whole cord blood samples of 4 mL or less within a few hours after delivery. Samples were kept at room temperature before and during processing. Red blood cells were removed by sedimentation with dextran and subsequent centrifugation typically yielding less than 2 ml of plasma containing white blood cells and platelets. Although previously we isolated CB ECFCs from the mononuclear cell fraction (21), we found that it does not result in a higher ECFC yield compared to unfractionated white blood cells in small size blood samples (data not shown). Using Vecstem^TM^ growth media (optimized for sustained growth of human ECFCs), we obtained ECFC clones from 8 of 13 cord blood samples (61%). Two donor samples yielded a single ECFC clone each, three samples—two clones, two samples—three clones, and one sample—five clones. Each clone is currently preserved at passage 3 in liquid nitrogen.

**Figure 1:**
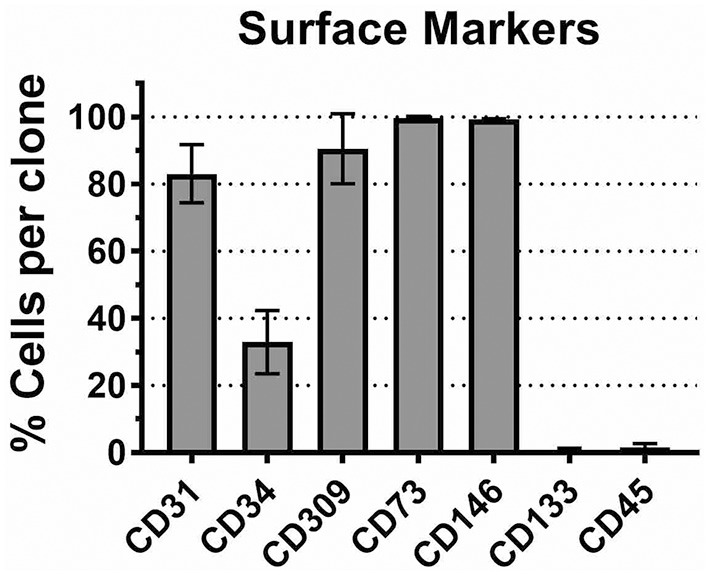
CD marker expression profile in CB ECFCs used in this study. Eight ECFC clones were stained with antibody specific to CD markers as described in Methods. The mean number of cells expressing corresponding antigen in each ECFC clone is plotted ± SD (*n* = 8).

For this study, we arbitrarily selected 8 ECFC clones representing 4 donors (2 clones per donor). Each ECFC clone was characterized by FACS analysis. Although, there is no a definitive molecular marker for ECFCs, several surface antigens were previously reported to be expressed by ECFCs. Cells were expanded in Vecstem^TM^ to obtain the necessary number for surface staining for CD31 (PECAM), CD309 (VEGFR2), CD73 (ecto-5′-nucleotidase), and CD146 (MCAM). In each ECFC clone, 80–100% of cells expressed these molecules (Figure 1). We found miniscule expression of stem and progenitor cell marker CD133 (prominin-1) and moderate but variable expression of CD34, as has been previously reported for cultured ECFCs (50). Finally, we tested the ECFC clones for the expression of leukocyte common antigen CD45 and confirmed that it is absent in our cell preparations (50). Thus, we can summarize the ECFC phenotype as CD31^+^/CD73^+^/CD146^+^/CD309^+^/CD133^−^/CD45^−^/CD34^var^.

### Generation of Concentration-Response Curves

Each ECFC clone was exposed to all eight chemicals. We seeded all eight ECFC clones at the same time in four 384-well plates (2 clones per plate). After an overnight incubation, cells were exposed to 9 titers of each chemical in duplicate (8 chemicals per plate); 48 h later, viable cells were stained and analyzed as described in the Methods. For each well, we obtained integrated optical density (OD) values as an indicator of cell viability. Raw data are provided in Supplemental Table 3. The OD value for each well was normalized to an untreated control (which represented 100% viability) and the normalized values were used to (1) generate concentration-response curves for each clone and each chemical and (2) to assess both the technical and biological variability of the data.

### Assessment of the Technical Reproducibility of the Assay (Day-To-Day Variability)

To compare concentration-effect curves generated for each chemical on different days, we initially exposed a polyclonal ECFC line CB002 (21) to four toxicants (CdCl_2_, NaAsO_2_, TBT, and menadione) at multiple concentrations. These data were used to compare runs performed on days 2 and 3 to the run performed on day 1, as described in Methods. Supplemental Figure 2 shows the concentration-response curves for each toxicant. Supplemental Figure 3 shows estimated β_2_(*t*) and β_3_(*t*) functions representing differences of the mean concentration-effect curves between day 2 vs. day 1 (panel A) and day 3 vs. day 1 (panel B), respectively. To assess the uncertainty of the estimates of β_2_(*t*) and β_3_(*t*), we conducted a permutation test with 1,000 repeats by permuting days for each toxicant. For each permutated data, we refitted the model 1 (see section Materials and Methods) to obtain 1,000 estimates of each coefficient function, from which we obtained the point-wise 95% confidence limits for β_2_(*t*) and β_3_(*t*) using the 2.5 and 97.5% percentiles to illustrate whether the point-wise difference between days is significant at the 5% level (Supplemental Figure 3).

Using the 1000 permutated data, we assessed the probability (proportion of repeats) of observing ≥the maximum ratio of |coefficient estimate|/standard error. For the global comparison of the concentration-effect curves obtained different days, the maximum ratio of |coefficient estimate|/standard error over t > 0 was calculated as 1.94 and 2.05, for day 2 vs. day 1 and day 3 vs. day 1, respectively, using the original data. The *p*-values were 0.74 and 0.64 for the comparison of day 2 vs. day 1 and day 3 vs. day 1, respectively, confirming that there is no strong evidence of significant differences between the results obtained on different days. These results indicate the high day-to-day reproducibility of our assay.

### Comparison of Individual Concentration-Response Curves

**Figure 2:**
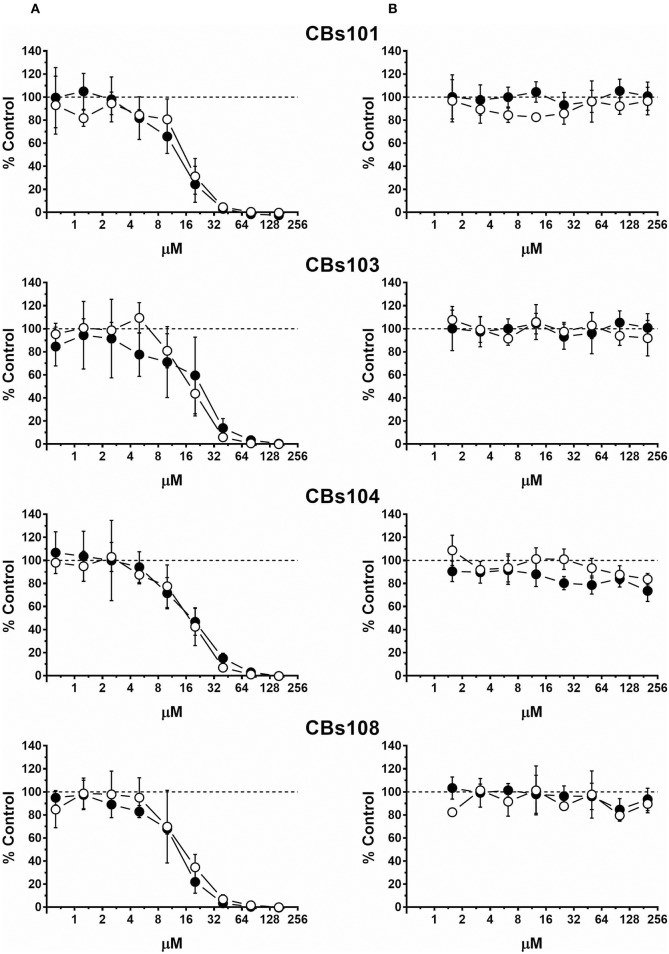
Concentration-response to cadmium and zinc. Cells were seeded and treated as described in Methods. Normalized OD values from three independent experiments were averaged and plotted against concentration of each chemical (μM). Error bars—standard deviation (SD, *n* = 3). Dashed line indicates 100% viability threshold. **(A)** CdCl_2_; **(B)** ZnCl_2_.

**Figure 3:**
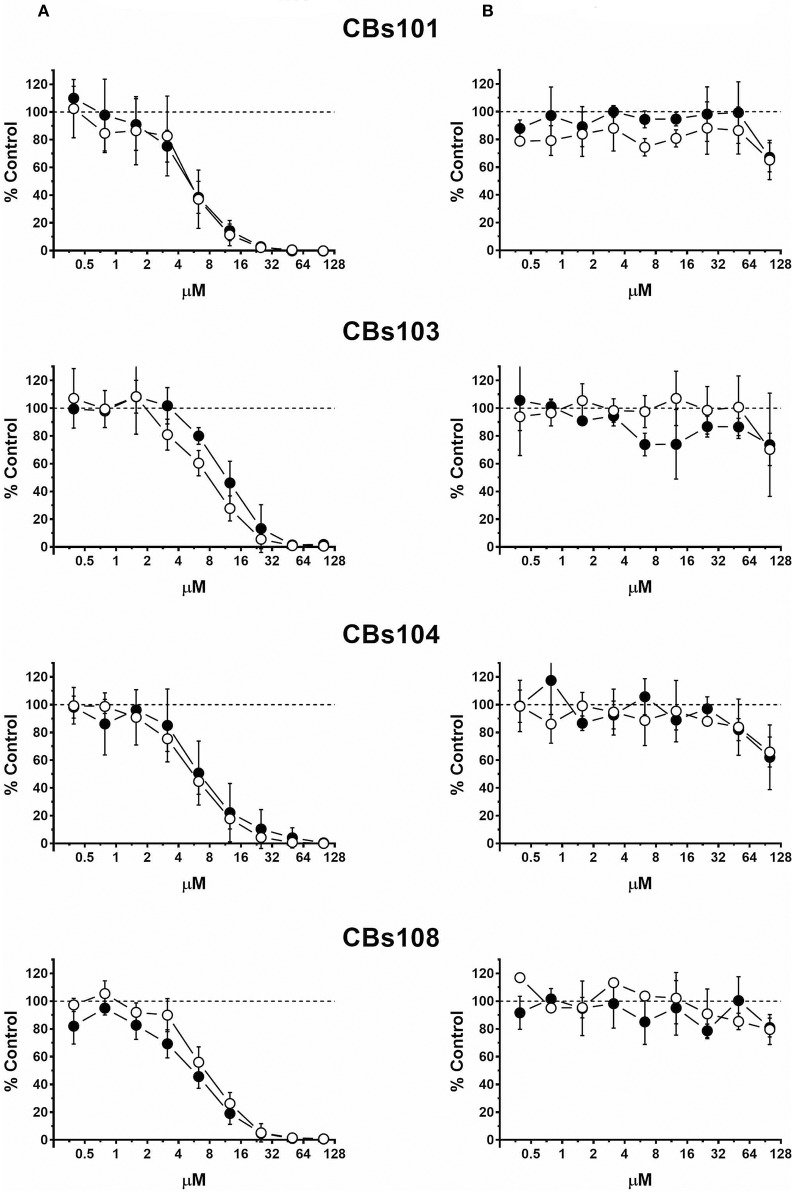
Concentration-response to arsenic chemicals. Cells were seeded and treated as described in Methods. Normalized OD values from three independent experiments were averaged and plotted against concentration of each chemical (μM). Error bars—standard deviation (SD, *n* = 3). Dashed line indicates 100% viability threshold. **(A)** NaAsO_2_(III); **(B)** Na_2_HAsO_4_(V).

**Figure 4:**
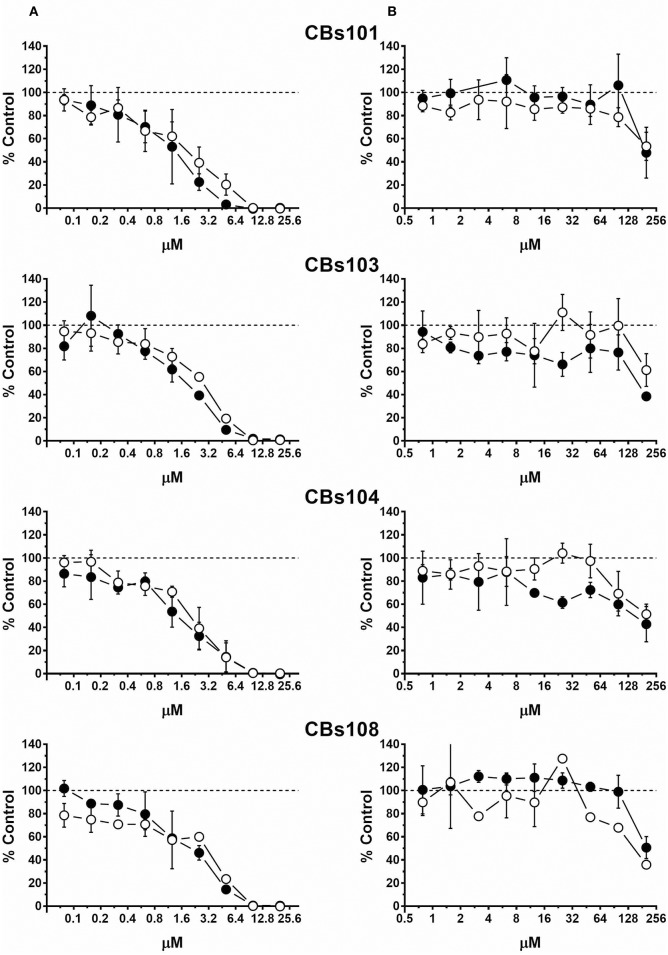
Concentration-response to tin-containing chemicals. Cells were seeded and treated as described in Methods. Normalized OD values from three independent experiments were averaged and plotted against concentration of each chemical (μM). Error bars—standard deviation (SD, *n* = 3). Dashed line indicates 100% viability threshold. **(A)** TBT; **(B)** SnCl_2_.

**Figure 5:**
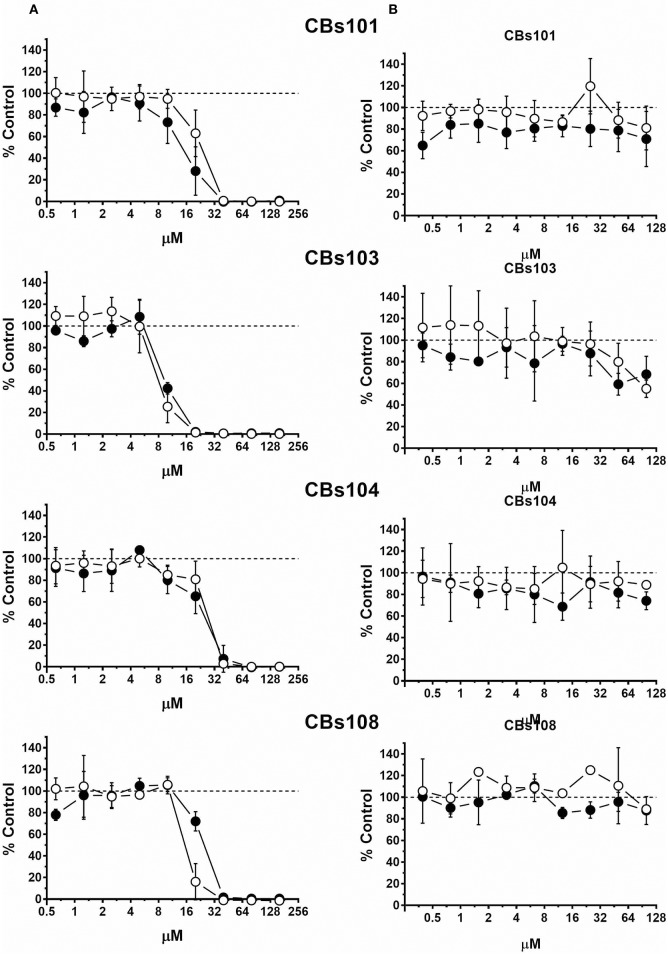
Differential effect of menadione and phytonadione on ECFCs. Cells were seeded and treated as described in Methods. Normalized OD values from three independent experiments were averaged and plotted against concentration of each chemical (μM). Error bars—standard deviation (SD, *n* = 3). Dashed line indicates 100% viability threshold. **(A)** Menadione; **(B)** Phytonadione.

Concentration-response curves for each ECFC clone using normalized data are presented in Figures 2–**5**. Each figure shows the responses of the all eight ECFCs clones to a specific pair of toxic/nontoxic chemicals.

#### Cadmium and Zinc

Figure 2 demonstrates effect of (A) cadmium (four *left* panels) and (B) zinc (four *right* panels) on cell viability in each ECFC clone. Each individual panel shows the results obtained by the treatment of two ECFC clones (presented as white and black circles) from the same donor. There is a striking similarity in how all eight clones derived from four donors respond to each chemical: with cadmium being much more toxic than zinc. In our assay, cadmium was not cytotoxic at <3 μM, while at 50 μM it killed practically 100% cells in each ECFC clone. Zinc did not affect viability at concentrations up to 200 μM but was cytotoxic at 400 μM.

#### Arsenic

In Figure 3, the comparison of trivalent and pentavalent arsenic compounds demonstrates that pentavalent arsenic had no effect on ECFCs viability within the concentration range tested, while trivalent counterpart was highly toxic: 50 μM of Na_2_HAsO_4_ and NaAsO_2_ killed 0 and 100% of cells in each ECFC clone, respectively. For both toxic and nontoxic forms of arsenic, each of eight ECFC clones demonstrated very similar concentration-response profile.

#### Tin-Containing Chemicals

Figure 4 demonstrates that TBT was the most toxic chemical among those tested. TBT at 10 μM was 100% toxic and even 1 μM was highly toxic. In contrast, inorganic tin, SnCl_2_ induced lower toxicity compared to TBT toxicity, as 200 μM was needed to induce a ~50% reduction of cell viability.

#### Vitamins K1 and K3

As expected, natural vitamin K1 (phytonadione) was not toxic to ECFCs at all concentrations tested (Figure 5). In stark contrast, K3 (menadione), a synthetic vitamin K analog decreased ECFC viability in a concentration-dependent manner. At 50 μM, 100% of cells were dead in each of the ECFC clones. However, inspection of the concentration-response curves revealed donor-specific differences in the responses to menadione within the 5–40 μM concentration range. For example, 100% of cells were viable in the CBs108 clones at 10 μM of menadione but a variable percentage of cells were viable in ECFC clones derived from other donors with only <50% live in CBs103 clones. These data indicate that some chemicals may exert a donor-specific toxicity toward ECFCs.

### Comparison of Chemicals in Toxic/Nontoxic Pairs

**Figure 6:**
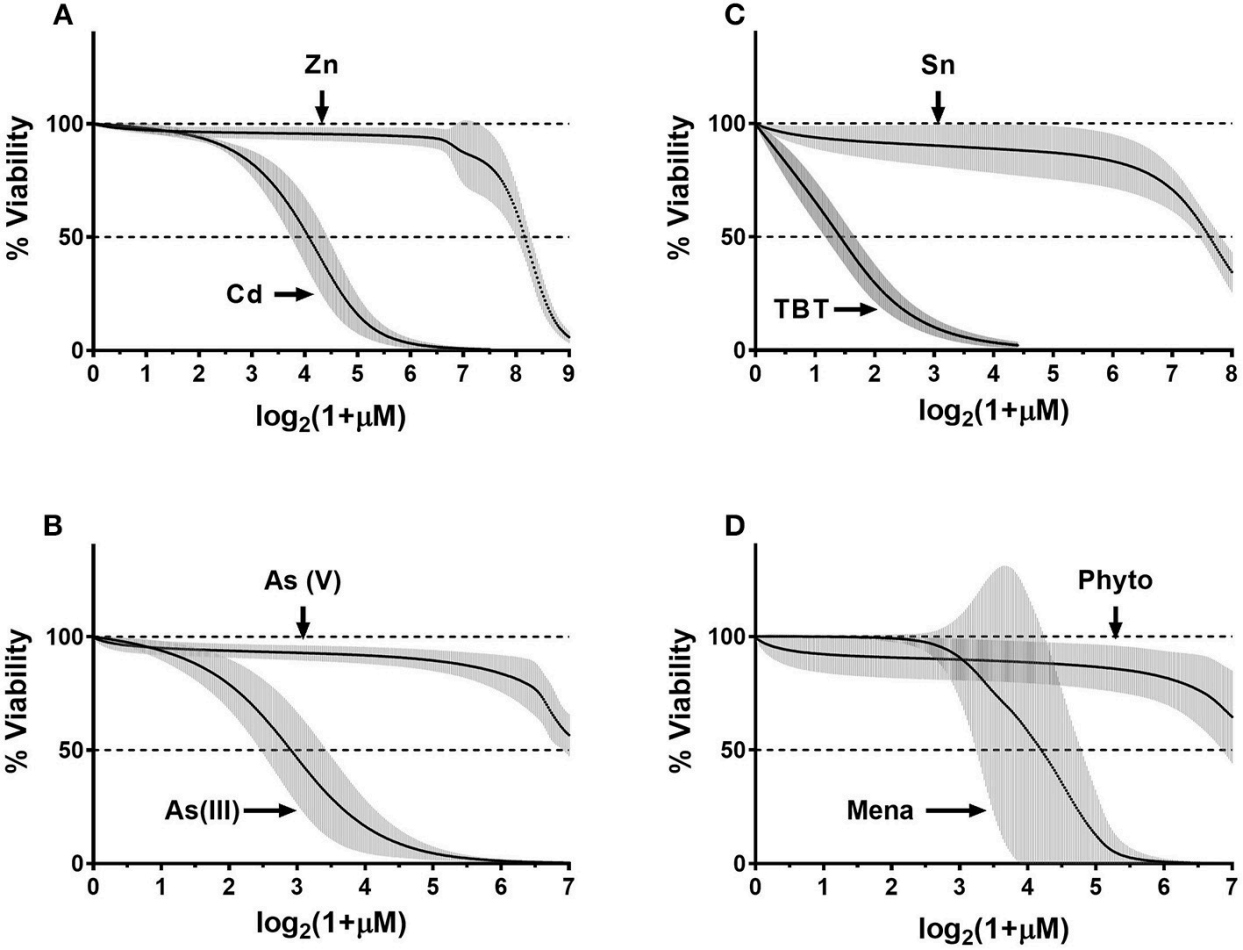
Composite fit-curve data for all donors. Data were fit as described in the Methods. We obtained a fit curve for each donor (*n* = 4) for each of the eight chemicals. Each donors' fit-curve has 300 data-points plotted on x-axis vs. % viability on the y-axis. Point-by-point mean values were calculated and plotted for four donors for each chemical (black dots) and 95% confidence interval (95%CI) was calculated for each data point (gray error bars).**(A)** CdCl_2_- ZnCl_2_; **(B)** As(III)-As(V); **(C)** TBT-SnCl_2_; **(D)** Menadione-Phytonadione.

To generalize donor-specificity and chemical-specificity of donors' responses, we fitted the normalized data as described in Methods. Overall, we generated thirty-two fit-curves: one curve per chemical (eight total) per donor (four total). For each pair of toxic/nontoxic chemicals, we plotted the fitted curves for all four donors on the same graph. Figure 6 demonstrates that in each pair all donors correctly differentiated the more toxic chemical from to its nontoxic counterpart (e.g., Cd vs. Zn). Donors exhibited similar responses with a relatively narrow distribution within their respective 95% confidence intervals (95%CI, gray areas in Figure 6). Only menadione caused a highly variable response, as reflected in Figure 6, panel D. In addition, the fitted concentration-response curves for Zn, As(III), SnCl_2_, and phytonadione showed some variability within certain concentration ranges for each chemical but it was not clear how significant was the donor-related influence on data distribution. Therefore, we conducted a statistical analysis to assess the contribution of both biological (within-and between-donor) and technical (day-to-day) reproducibility to the overall variability of ECFC responses.

Formal evaluation of the proportional contribution of both biological (between-donors and between all ECFC clones) and technical (day-to-day between clones) variabilities is described in Methods. In Table 1, we present calculated total, between-donor (column 1), between-clone (column 2), and within-clone (technical, or day-to-day, column 3) variations in terms of sum of squared deviations for the four most toxic chemicals. The calculated variation values can be used to estimate the variability for each examined cytotoxic effect (i.e., for 10, 20, 30%, etc.). The numbers in bold indicate variation that contributed the most to the total variation.

**Table 1:** Relative contribution of biological and technical variability in total variability.

**Chemical**	**Inhibitory concentration (IC)/% viability**	**Variation (% of total variation) between:**		
		**1. Donors**	**2. Clones**	3. Days^*^
CdCl_2_	IC10/90	44.82 (11.6)	23.17 (6.0)	**318.68 (82.4)**
	IC20/80	49.81 (10.2)	27.05 (5.6)	**409.96 (84.2)**
	IC30/70	54.87 (10.2)	25.90 (4.8)	**455.62 (84.9)**
	IC40/60	65.05 (11.7)	22.48 (4.0)	**469.36 (84.3)**
	IC50/50	84.83 (15.1)	20.51 (3.7)	**456.26 (81.2)**
	IC60/40	120.98 (21.1)	28.20 (4.9)	**424.30 (74.0)**
	IC70/30	189.38 (28.8)	68.41 (10.4)	**398.68 (60.7)**
	IC80/20	342.03 (32.1)	228.40 (21.4)	**494.96 (46.5)**
	IC90/10	874.86 (23.4)	1135.08 (30.4)	**1726.37 (46.2)**
NaAsO_2_	IC10/90	10.89 (23.6)	10.33 (22.3)	**25.01 (54.1)**
	IC20/80	19.97 (26.2)	17.55 (23.0)	**38.76 (50.8)**
	IC30/70	29.91 (28.4)	23.94 (22.7)	**51.39 (48.8)**
	IC40/60	41.74 (30.3)	30.16 (21.9)	**65.79 (47.8)**
	IC50/50	56.78 (31.7)	36.64 (20.5)	**85.49 (47.8)**
	IC60/40	77.44 (32.4)	43.89 (18.4)	**117.34 (49.2)**
	IC70/30	109.05 (32.0)	52.83 (15.5)	**178.62 (52.5)**
	IC80/20	166.91 (29.7)	65.79 (11.7)	**328.87 (58.6)**
	IC90/10	324.56 (24.0)	94.14 (6.9)	**935.97 (69.1)**
TBT	IC10/90	0.13 (9.4)	0.13 (9.4)	**1.13 (81.3)**
	IC20/80	0.29 (11.5)	0.24 (9.5)	**2.00 (79.1)**
	IC30/70	0.49 (13.4)	0.41 (11.2)	**2.76 (75.4)**
	IC40/60	0.76 (15.4)	0.66 (13.4)	**3.52 (71.3)**
	IC50/50	1.16 (17.5)	1.09 (16.5)	**4.36 (66.0)**
	IC60/40	1.85 (19.8)	2.00 (21.5)	**5.47 (58.7)**
	IC70/30	3.38 (22.1)	4.47 (29.2)	**7.46 (48.7)**
	IC80/20	8.26 (22.9)	**13.79 (38.3)**	**13.99 (38.8)**
	IC90/10	38.98 (20.9)	**79.77 (42.8)**	67.58 (36.3)
Menadione	IC10/90	**314.56 (43.3)**	191.48 (26.3)	221.03 (30.4)
	IC20/80	**411.48 (50.6)**	191.74 (23.6)	209.48 (25.8)
	IC30/70	**501.9 (56.5)**	186.11 (21.0)	200.20 (22.5)
	IC40/60	**598.84 (61.6)**	178.47 (18.4)	194.65 (20.0)
	IC50/50	**712.2 (66.1)**	170.08 (15.8)	194.52 (18.1)
	IC60/40	**856.44 (70.1)**	161.89 (13.2)	203.78 (16.7)
	IC70/30	**1059.89 (73.2)**	156.18 (10.8)	232.57 (16.1)
	IC80/20	**1397.55 (74.6)**	161.73 (8.6)	314.02 (16.8)
	IC90/10	**2186.21 (71.7)**	236.22 (7.7)	625.89 (20.5)

* *Variability between data obtained on 3 different days for the same clones (within-clones variability). Values that contributed the most to the variability are shown in bold*.

## Discussion

We verified the identity of the isolated cell populations by FACS analysis of cell-surface antigens; the results indicated that the typical endothelial cell markers (i.e., CD31, CD309, CD73, and CD146) were present in 80 to 100% of cells in each ECFC clone (Figure 1). Only 20–30% of cells in each clone expressed CD34, a hematopoietic stem cell antigen; a reduced expression of this antigen is thought to be characteristic to the cultured (probably, more mature) endothelial progenitor cells (50). Also, the ECFC clones expressed very low level of CD133, a progenitor cell marker, and CD45, a leukocyte common antigen. Overall, the observed CD antigen profile in our ECFC clones corresponds to previously reported data on ECFC (15). As a sub-population of endothelial progenitor cells, ECFCs have been the focus of considerable efforts over the past decade to characterize them both phenotypically and functionally. Although the origin of these cells (bone marrow vs. vessel walls) remains a topic of debate (50), there is a consensus about the key role these cells play in neovascularization (51, 52). Therefore, an exposure of fetal/neonatal ECFCs to toxic chemicals can potentially lead to a functional impairment of the vasculature.

In terms of the chemical concentrations used in our study, exposure to cadmium, arsenic, tin, and menadione has been shown to damage endothelial cells and have been associated with a variety of pathological conditions—from atherosclerosis and hypertension to cardio- and nephropathy to cancer. Blood levels of Cd at 10–100 nM (53) and arsenic at 30 nM (54) occur in the general population. Although, these numbers are significantly lower than the micromolar concentrations used in our study, in certain pathological conditions various tissues tend to accumulate high levels of both Cd and As. Thus, an average Cd concentration of 7 μM was found in the medial layer of the abdominal aorta in smokers (55) and in lungs of emphysema patients (56). Also, the concentration of As can increase two–three-fold in blood after chronic exposure, although there is not data as to As valency (54). Zinc concentrations in normal human plasma have been reported at 15 μM (57) and 70 μM (54), which is well within the concentrations used in our study. Wide variations in the levels of inorganic tin were observed among individuals and in different tissues, with an average 17 mg of tin per human body, two-thirds of which is associated with bones with the remaining largely found in the kidney, liver, and lung. In one study, 70% of human blood samples contained organic tin compounds, including an average 56 nM TBT except two persons that had 7- and 14-times higher levels (58), which is close to the toxic TBT concentrations found in this work. While Cd, As, and TBT are environmental pollutants, the Vitamin K derivatives phytonadione (K1), and menadione (K3) are important physiological constituents, which are also used for the treatment, control, and prevention of blood disorders characterized by Vitamin K or prothrombin deficiencies. The concentration of Vitamin K1 in adult plasma is generally within the 0.3–3.0 nM range, while infant cord plasma has been reported to contain 16 pM, although even 1,000-times higher supraphysiological levels of Vitamin K1 is well tolerated by preterm neonates (59) and there is no evidence to derive a tolerable upper intake level (60). Previously, 0.5–2 μM menadione blood level was observed in a clinical setting (37, 61) and in a recent study, 20 nM menadione was found in the blood of volunteers after ingestion of 10 mg menadione sodium bisulfite tablets (62). Thus, chemical concentrations used in our study are relevant to the physiological levels found in humans.

Initially, we assessed assay reproducibility using a polyclonal cord blood derived ECFC line exposed to developmental and cardiovascular toxicants CdCl_2_, NaAsO_2_, TBT, and menadione and found that the variability between independent experiments performed on separate days was low (Supplemental Figure 3). Next, we tested the extent of cytotoxicity induced by the same toxicants and their four relatively nontoxic chemical counterparts in eight cord-blood derived ECFC clones, with 2 clones from each of 4 donors (see Methods and Supplemental Figure 1). The resulting concentration-response data were used to evaluate sources of variability, that is the relative contribution of (a) day-to-day (within clones assayed on three different days) variability, (b) variability between clones derived from the same donor, and (c) variability between donors. For this analysis, we calculated how the between-donor, between-clone, and between-day (within clone) variations contribute to the total variation at selected levels of cytotoxicity. In Table 1, we present calculations for nine levels of cytotoxicity/viability: from an IC10 (90% viable cells) to an IC90 (10% viable cells). When we compared variations at the same level of cell viability within the 10–90% cytotoxicity range for CdCl_2_, NaAsO_2_, and TBT, between-donor variations were either equal to or slightly higher than between-clone variations, with the exception for TBT at 10 and 20% viability levels (Table 1, compare variations across the same rows). For CdCl_2_ and TBT, the variation between the ECFC clones tended to increase with a reduction of cell viability but it was the opposite for NaAsO_2_. For these three toxicants, the observed differences between donors and clones were too small compared to the difference between the experiments performed on different days and the number of subjects is too few and therefore, no conclusions can be drawn at this point in regard to whether or not individual differences in susceptibility are likely to occur. However, in contrast, for menadione, the contribution of between-donor variations was much greater than that observed for the between-clone variances. At an IC10, 43.3% of the total variation was due to between-donor variation while only 26.3% was due to between-clone variation (Table 1). As the viability decreased in cultures exposed to higher menadione concentrations, the input from the between-donor variation increased (>70% at >IC60), while between-clone variation steadily decreased to 7.7% at an IC90. The proportion of day-to-day variation fluctuated between 15 and 30%. These data suggest that an individual clone is likely to represent a donor's response to a toxicant and that at least for some chemicals, cell-based assays can distinguish more sensitive from less sensitive individuals. The mechanism by which ECFCs from different donors vary in their response to menadione is a subject for further research.

In Table 2, we present the calculated IC10 and IC50 values for each ECFC clone and each of the four toxic compounds. The IC10 values for CdCl_2_, NaAsO_2_, and TBT varied between individual ECFC clones from 4.2–9.3 μM, 1.1–5.0 μM, and 0.2–0.5 μM, respectively. The corresponding IC50 values are 12.3–18.0 μM, 4.9–15.1 μM, and 1.2–2.3 μM, respectively. For menadione, the IC10 and IC50 values are 5.6–19.0 μM and 8.0–26.3 μM, respectively. Although the ranges of values are wider for menadione, a comparison of IC50 or IC10 values does not provide insight into the variability of ECFC responses and specifically, the extent of between-donor variability in response to menadione vs. the much more uniform response to CdCl_2_, NaAsO_2_, and TBT (as demonstrated in Figures 2–4, 5, respectively).

**Table 2:** IC10 and IC50 (μM) for each ECFC clone derived from fitted curves.

**ECFC clone**	***n***	**CdCl**_****2****_		**NaAsO**_****2****_		**TBT**		**Menadione**	
		**IC10 ± SD**	**IC50 ± SD**	**IC10 ± SD**	**IC50 ± SD**	**IC10 ± SD**	**IC50 ± SD**	**IC10 ± SD**	**IC50 ± SD**
CBs101-1P6	3	7.2 ± 2.8	15.4 ± 3.8	2.0 ± 2.0	4.9 ± 2.4	0.2 ± 0.2	1.6 ± 0.9	14.2 ± 4.3	21.7 ± 3.5
CBs101-2P6	3	4.5 ± 3.7	12.3 ± 4.6	1.9 ± 1.2	5.0 ± 1.5	0.4 ± 0.6	1.2 ± 0.8	6.2 ± 4.1	14.2 ± 4.7
CBs103-1P6	3	9.3 ± 3.9	17.8 ± 4.6	2.6 ± 1.5	7.6 ± 2.0	0.5 ± 0.4	2.3 ± 0.3	5.6 ± 1.5	8.0 ± 1.4
CBs103-2P6	2	8.6 ± 11.7	18.0 ± 15.1	5.0 ± 1.1	12.1 ± 3.6	0.4 ± 0.2	1.7 ± 0.4	6.5 ± 0.9	9.3 ± 0.5
CBs104-1P6	3	7.5 ± 4.4	16.7 ± 5.0	1.7 ± 1.0	5.5 ± 0.2	0.3 ± 0.1	1.9 ± 0.5	19.0 ± 7.3	26.3 ± 5.3
CBs104-2P6	3	5.5 ± 4.3	17.2 ± 3.4	2.3 ± 1.7	7.5 ± 4.7	0.2 ± 0.1	1.3 ± 0.2	11.8 ± 4.1	22.4 ± 5.3
CBs108-1P6	2	6.4 ± 5.4	14.4 ± 6.1	2.6 ± 0.2	7.5 ± 1.2	0.2 ± 0.1	1.6 ± 0.7	12.7 ± 0.9	16 ± 2.4
CBs108-2P6	3	4.2 ± 1.1	12.3 ± 1.6	1.1 ± 0.5	5.3 ± 1.4	0.3 ± 0.2	1.7 ± 0.4	16.4 ± 1.3	23.1 ± 1.3

*SD, standard deviation*.

These experiments also aimed to determine whether these ECFCs could correctly distinguish between known toxic chemicals and their nontoxic counterparts, so that these cells could be used to rank chemicals by their toxicity profile. Among the chemicals tested, there was an obvious difference in response to the four toxic chemicals and their relatively nontoxic counterparts. IC10 values can be used to indicate the chemical concentration at which a significant cytotoxic response begins to occur in this cell population. Based on average IC10 values, which were calculated using IC10 values for individual ECFC clones (Table 1), the most toxic chemical was TBT (IC10 = 0.3 ± 0.11 μM, mean and standard deviation) followed by NaAsO_2_ (IC10 = 2.4 ± 1.16 μM), CdCl_2_ (IC10 = 6.7 ± 1.85 μM), and menadione (IC10 = 11.5 ± 5.03 μM). Due to the lack of a robust cytotoxic response, IC10 values for the nontoxic counterparts (although obviously higher) could not be accurately determined over the concentration ranges tested. These data demonstrate that the ECFCs respond as expected to toxic and relatively nontoxic chemicals.

This study sets the foundation for the more comprehensive studies that are needed to convincingly determine whether donor-derived clonal populations of cells can represent donor-specific characteristics in epidemiological studies. This basic question needs to be answered so that cell-based assays can be used instead of animal models to (a) estimate individual variability of *human* responses to environmental toxicants and drugs, and (b) predict individual vulnerability to the harmful effects of a specific toxicant/drug. Such information can be useful for example in ranking chemicals with respect to the proportion of the population that might be vulnerable to a chemical at certain concentrations. Such ranking can be important, considering that humans are exposed to an increasing number of industrial, agricultural, and household chemicals (63–65). Recently, a similar approach was used to assess the feasibility of using a population-based iPSC-derived cardiomyocytes as an animal replacement experimental model (66). The authors found reproducible donor-specific differences in baseline function and drug-induced effects and concluded that the model platform could be used to rapidly screen drugs and chemicals for inter-individual variability in cardiotoxicity.

Our results demonstrate the potential utility of using this platform for evaluating the effect of intrinsic (i.e., genetic/epigenetic) sensitivity of cord-blood derived ECFC, representing the response of neonatal populations to drugs and environmental toxicants (11). ECFC-based assays may serve as a platform to evaluate individual responses to various toxicants but there is a need to expand the number of donors as well as the breadth of tested chemicals to further characterize the reliability and relevance of the presented methodology.

## Author Contributions

DI and AK came up with the idea, designed experiments, analyzed data, and wrote the manuscript. RT analyzed data and wrote the manuscript. DF, MV, and JL ran experiments and analyzed data, RL analyzed data. CG enrolled participants and collected blood.

### Conflict of Interest Statement

DF, RT, and AK are employed by Creative Scientist, Inc. MV and JL are employed by ZenBio, Inc. The remaining authors declare that the research was conducted in the absence of any commercial or financial relationships that could be construed as a potential conflict of interest.

## References

[1] Chiu WA Rusyn I. Advancing chemical risk assessment decision-making with population variability data: challenges and opportunities. Mamm Genome (2018) 29:182–9. 10.1007/s00335-017-9731-6 29299621 PMC5849521

[2] Roden DM George AL Jr. The genetic basis of variability in drug responses. Nat Rev Drug Discov. (2002) 1:37–44. 10.1038/nrd705 12119608

[3] Zeise L Bois FY Chiu WA Hattis D Rusyn I Guyton KZ. Addressing human variability in next-generation human health risk assessments of environmental chemicals. Environ Health Perspect. (2012) 121:23–31. 10.1289/ehp.1205687 23086705 PMC3553440

[4] The Complex Trait Consortium The Collaborative Cross, a community resource for the genetic analysis of complex traits. Nat Genet. (2004) 36:1133–7. 10.1038/ng1104-1133 15514660

[5] Harrill AH McAllister KA. New rodent population models may inform human health risk assessment and identification of genetic susceptibility to environmental exposures. Environ Health Perspect. (2017) 125:086002. 10.1289/EHP1274 28886592 PMC5783628

[6] French JE Gatti DM Morgan DL Kissling GE Shockley KR Knudsen GA. Diversity outbred mice identify population-based exposure thresholds and genetic factors that influence benzene-induced genotoxicity. Environ Health Perspect. (2015) 123:237–45. 10.1289/ehp.1408202 25376053 PMC4348743

[7] Dornbos P LaPres JJ. Incorporating population-level genetic variability within laboratory models in toxicology: from the individual to the population. Toxicology (2018) 395:1–8. 10.1016/j.tox.2017.12.007 29275117 PMC5801153

[8] Lock EF Abdo N Huang R Xia M Kosyk O O'Shea SH. Quantitative high-throughput screening for chemical toxicity in a population-based *in vitro* model. Toxicol Sci. (2012) 126:578–88. 10.1093/toxsci/kfs023 22268004 PMC3307611

[9] Abdo N Wetmore BA Chappell GA Shea D Wright FA Rusyn I. *In vitro* screening for population variability in toxicity of pesticide-containing mixtures. Environ Int. (2015) 85:147–55. 10.1016/j.envint.2015.09.012 26386728 PMC4773193

[10] The NIEHS-NCATS-UNC DREAM Toxicogenetics Collaboration Eduati F Mangravite LM Wang T Tang H Bare JC Prediction of human population responses to toxic compounds by a collaborative competition. Nat Biotechnol. (2015) 33:933–40. 10.1038/nbt.3299 26258538 PMC4568441

[11] Il'yasova D Kloc N Kinev A. Cord blood cells for developmental toxicology and environmental health. Front Public Health (2015) 3:265. 10.3389/fpubh.2015.00265 26697419 PMC4668287

[12] Urbich C Dimmeler S. Endothelial progenitor cells: characterization and role in vascular biology. Circ Res. (2004) 95:343–53. 10.1161/01.RES.0000137877.89448.78 15321944

[13] Ingram DA. Unresolved questions, changing definitions, and novel paradigms for defining endothelial progenitor cells. Blood (2005) 106:1525–31. 10.1182/blood-2005-04-1509 15905185

[14] Hirschi KK Ingram DA Yoder MC. Assessing identity, phenotype, and fate of endothelial progenitor cells. Arterioscler Thromb Vasc Biol. (2008) 28:1584–95. 10.1161/ATVBAHA.107.155960 18669889 PMC5244813

[15] Yoder MC Human endothelial progenitor cells. Cold Spring Harb Perspect Med. (2012) 2:a006692 10.1101/cshperspect.a006692 22762017 PMC3385946

[16] Palis J McGrath KE Kingsley PD. Initiation of hematopoiesis and vasculogenesis in murine yolk sac explants. Blood (1995) 86:156–163. 7795222

[17] Díaz-Flores L Gutiérrez R García-Suárez MP Sáez FJ Gutiérrez E Valladares F. Morphofunctional basis of the different types of angiogenesis and formation of postnatal angiogenesis-related secondary structures. Histol Histopathol. (2017) 32:1239–79. 10.14670/HH-11-923 28762232

[18] Gerecht-Nir S Osenberg S Nevo O Ziskind A Coleman R Itskovitz-Eldor J Vascular development in early human embryos and in teratomas derived from human embryonic stem cells1. Biol Reprod. (2004) 71:2029–36. 10.1095/biolreprod.104.031930 15317687

[19] Gumina DL Su EJ. Endothelial progenitor cells of the human placenta and fetoplacental circulation: a potential link to fetal, neonatal, and long-term health. Front Pediatr. (2017) 5:41. 10.3389/fped.2017.00041 28361046 PMC5350128

[20] O'Neill CL McLoughlin KJ Chambers SEJ Guduric-Fuchs J Stitt AW Medina RJ. The vasoreparative potential of endothelial colony forming cells: a journey through pre-clinical studies. Front Med. (2018) 5:273. 10.3389/fmed.2018.00273 30460233 PMC6232760

[21] Kinev AV Levering V Young K Ali-Osman F Truskey GA Dewhirst MW. Endothelial colony forming cells (ECFCs) as a model for studying effects of low-dose ionizing radiation: growth inhibition by a single dose. Cancer Invest. (2013) 31:359–64. 10.3109/07357907.2013.789903 23621632 PMC3754852

[22] Ludlow JW Kinev A VanKanegan M Ben Buehrer Trotta N Basu J Chapter 5 toxicological risk assessment - proposed assay platform using stem and progenitor cell differentiation in response to environmental toxicants. In: Sherley JL, editor. Human Stem Cell Toxicology. Cambridge, UK: The Royal Society of Chemistry (2016). p. 94–123.

[23] Solenkova NV Newman JD Berger JS Thurston G Hochman JS Lamas GA. Metal pollutants and cardiovascular disease: mechanisms and consequences of exposure. Am Heart J. (2014) 168:812–22. 10.1016/j.ahj.2014.07.007 25458643 PMC4254412

[24] Razagui IB-A Ghribi I. Maternal and neonatal scalp hair concentrations of zinc, copper, cadmium, and lead. Biol Trace Elem Res. (2005) 106:1–27. 10.1385/BTER:106:1:001 16037607

[25] Lukkhananan P Thawonrachat N Srihirun S Swaddiwudhipong W Chaturapanich G Vivithanaporn P. Endothelial dysfunction in subjects with chronic cadmium exposure. J Toxicol Sci. (2015) 40:605–13. 10.2131/jts.40.605 26354377

[26] Bernhard D Rossmann A Henderson B Kind M Seubert A Wick G. Increased serum cadmium and strontium levels in young smokers: effects on arterial endothelial cell gene transcription. Arterioscler Thromb Vasc Biol. (2006) 26:833–8. 10.1161/01.ATV.0000205616.70614.e5 16439709

[27] Simeonova P. Arsenic and atherosclerosis. Toxicol Appl Pharmacol. (2004) 198:444–9. 10.1016/j.taap.2003.10.018 15276425

[28] McCollum CW Hans C Shah S Merchant FA Gustafsson J-Å Bondesson M. Embryonic exposure to sodium arsenite perturbs vascular development in zebrafish. Aquat Toxicol. (2014) 152:152–63. 10.1016/j.aquatox.2014.04.006 24768856

[29] Botelho G Bernardini C Zannoni A Ventrella V Bacci ML Forni M. Effect of tributyltin on mammalian endothelial cell integrity. Comp Biochem Physiol Toxicol Pharmacol CBP (2015) 176–177:79–86. 10.1016/j.cbpc.2015.07.012 26256121

[30] Antizar-Ladislao B. Environmental levels, toxicity and human exposure to tributyltin (TBT)-contaminated marine environment. a review. Environ Int. (2008) 34:292–308. 10.1016/j.envint.2007.09.005 17959247

[31] Oyanagi K Tashiro T Negishi T. Cell-type-specific and differentiation-status-dependent variations in cytotoxicity of tributyltin in cultured rat cerebral neurons and astrocytes. J Toxicol Sci. (2015) 40:459–68. 10.2131/jts.40.459 26165642

[32] Ogata R Omura M Shimasaki Y Kubo K Oshima Y Aou S. Two-generation reproductive toxicity study of tributyltin chloride in female rats. J Toxicol Environ Health A (2001) 63:127–44. 10.1080/15287390151126469 11393799

[33] EFSA Panel on Additives and Products or Substances used in Animal Feed Scientific Opinion on the safety and efficacy of vitamin K3 (menadione sodium bisulphite and menadione nicotinamide bisulphite) as a feed additive for all animal species. EFSA J. (2014) 12:3532 10.2903/j.efsa.2014.3532 PMC13159841 42125110

[34] Ivanova D Zhelev Z Getsov P Nikolova B Aoki I Higashi T. Vitamin K: redox-modulation, prevention of mitochondrial dysfunction and anticancer effect. Redox Biol. (2018) 16:352–8. 10.1016/j.redox.2018.03.013 29597144 PMC5953218

[35] Criddle DN Gillies S Baumgartner-Wilson HK Jaffar M Chinje EC Passmore S. Menadione-induced reactive oxygen species generation via redox cycling promotes apoptosis of murine pancreatic acinar cells. J Biol Chem. (2006) 281:40485–92. 10.1074/jbc.M607704200 17088248

[36] Warren MC Bump EA Medeiros D Braunhut SJ. Oxidative stress–induced apoptosis of endothelial cells. Free Radic Biol Med. (2000) 29:537–47. 10.1016/S0891-5849(00)00353-1 11025197

[37] Lee J-Y Lee M-Y Chung S-M Chung J-H. Menadione-induced vascular endothelial dysfunction and its possible significance. Toxicol Appl Pharmacol. (1999) 161:140–5. 10.1006/taap.1999.8795 10581207

[38] Tang L Qiu R Tang Y Wang S. Cadmium–zinc exchange and their binary relationship in the structure of Zn-related proteins: a mini review. Metallomics (2014) 6:1313. 10.1039/C4MT00080C 24806548

[39] Cousins RJ Blanchard RK Moore JB Cui L Green CL Liuzzi JP. Regulation of Zinc Metabolism and Genomic Outcomes. J Nutr. (2003) 133:1521S−6S. 10.1093/jn/133.5.1521S 12730457

[40] Mocchegiani E Romeo J Malavolta M Costarelli L Giacconi R Diaz L-E. Zinc: dietary intake and impact of supplementation on immune function in elderly. AGE (2013) 35:839–60. 10.1007/s11357-011-9377-3 22222917 PMC3636409

[41] Hartwig A Asmuss M Ehleben I Herzer U Kostelac D Pelzer A. Interference by toxic metal ions with DNA repair processes and cell cycle control: molecular mechanisms. Environ Health Perspect. (2002) 110:797–9. 10.1289/ehp.02110s5797 12426134 PMC1241248

[42] Ratnaike RN. Acute and chronic arsenic toxicity. Postgrad Med J. (2003) 79:391–6. 10.1136/pmj.79.933.391 12897217 PMC1742758

[43] Nakamuro K Sayato Y. Comparative studies of chromosomal aberration induced by trivalent and pentavalent arsenic. Mutat Res Toxicol. (1981) 88:73–80. 10.1016/0165-1218(81)90091-4 7207493

[44] Styblo M Del Razo LM Vega L Germolec DR LeCluyse EL Hamilton GA. Comparative toxicity of trivalent and pentavalent inorganic and methylated arsenicals in rat and human cells. Arch Toxicol. (2000) 74:289–99. 11005674 10.1007/s002040000134

[45] Howe P Watts P Tin and Inorganic tin Compounds. Geneva: World Health Organization (2005).

[46] Solomon R Krishnamurty V. The effect of tributyltin chloride on vascular responses to atrial natriuretic peptide. Toxicology (1992) 76:39–47. 10.1016/0300-483X(92)90016-8 1335619

[47] Ramsay JO Functional Data Analysis. In: Balakrishnan N, editor. Encyclopedia of Statistical Sciences. American Cancer Society, Hoboken, NJ: John Wiley & Sons (2006). p. 9686.

[48] Boor C de A Practical Guide to Splines. New York, NY: Springer-Verlag (1978). Available online at: //www.springer.com/us/book/9780387953663 (Accessed October 1, 2018)

[49] Goldsmith J Scheipl F Huang L Wrobel J Gellar J Harezlak J Refund: Regression With Functional Data. (2018). Available online at: https://CRAN.R-project.org/package=refund (Accessed September 29, 2018)

[50] Huizer K Mustafa DAM Spelt JC Kros JM Sacchetti A. Improving the characterization of endothelial progenitor cell subsets by an optimized FACS protocol. PLOS ONE (2017) 12:e0184895. 10.1371/journal.pone.0184895 28910385 PMC5599045

[51] Wong WT Huang NF Botham CM Sayed N Cooke JP. Endothelial cells derived from nuclear reprogramming. Circ Res. (2012) 111:1363–75. 10.1161/CIRCRESAHA.111.247213 23104878 PMC3526979

[52] Yoder MC. Endothelial progenitor cell: a blood cell by many other names may serve similar functions. J Mol Med. (2013) 91:285–95. 10.1007/s00109-013-1002-8 23371317 PMC3704045

[53] Prozialeck WC Edwards JR Woods JM. The vascular endothelium as a target of cadmium toxicity. Life Sci. (2006) 79:1493–506. 10.1016/j.lfs.2006.05.007 16765992

[54] Buscemi S Vasto S Di Gaudio F Grosso G Bergante S Galvano F. Endothelial function and serum concentration of toxic metals in frequent consumers of fish. PLoS ONE (2014) 9:e112478. 10.1371/journal.pone.0112478 25401695 PMC4234466

[55] Abu-Hayyeh S Sian M Jones KG Manuel A Powell JT. Cadmium accumulation in aortas of smokers. Arterioscler Thromb Vasc Biol. (2001) 21:863–7. 10.1161/01.ATV.21.5.863 11348888

[56] Pääkkö P Kokkonen P Anttila S Kalliomäki PL. Cadmium and chromium as markers of smoking in human lung tissue. Environ Res. (1989) 49:197–207. 2753006 10.1016/s0013-9351(89)80065-9

[57] Cao J Cousins RJ. Metallothionein mRNA in monocytes and peripheral blood mononuclear cells and in cells from dried blood spots increases after zinc supplementation of men. J Nutr. (2000) 130:2180–7. 10.1093/jn/130.9.2180 10958810

[58] Kannan K Senthilkumar K Giesy JP Occurrence of butyltin compounds in human blood. Environ Sci Technol. (1999) 33:1776–9. 10.1021/es990011w

[59] Clarke P. Vitamin K prophylaxis for preterm infants. Early Hum Dev. (2010) 86:17–20. 10.1016/j.earlhumdev.2010.01.013 20106609

[60] Turck D Bresson J-L Burlingame B Dean T Fairweather-Tait S Heinonen M Dietary reference values for vitamin K. EFSA J. (2017) 15:e04780 10.2903/j.efsa.2017.4780 PMC7010012 32625486

[61] Akman SA Kusu F Takamura K Chlebowski R Block J. Differential pulse polarographic determination of plasma menadione. Anal Biochem. (1984) 141:488–93. 6496951 10.1016/0003-2697(84)90075-7

[62] Yuan T-F Wang S-T Li Y. Quantification of menadione from plasma and urine by a novel cysteamine-derivatization based UPLC–MS/MS method. J Chromatogr B (2017) 1063:107–11. 10.1016/j.jchromb.2017.08.026 28858751

[63] US EPA O TSCA Chemical Substance Inventory. Available online at: https://www.epa.gov/tsca-inventory#background (Accessed March 27, 2016)

[64] Judson R Richard A Dix DJ Houck K Martin M Kavlock R. The toxicity data landscape for environmental chemicals. Environ Health Perspect. (2009) 117:685–95. 10.1289/ehp.0800168 19479008 PMC2685828

[65] Collins FS Gray GM Bucher JR. Transforming environmental health protection. Science (2008) 319:906–7. 10.1126/science.1154619 18276874 PMC2679521

[66] Grimm F. A human population-based organotypic *in vitro* model for cardiotoxicity screening. ALTEX (2018) 441–52. 10.14573/altex.1805301 29999168 PMC6231908

