# Supplementary material for: Initial Assessment of Variability of Responses to Toxicants in Donor-Specific Endothelial Colony Forming Cells
Source: Front Public Health. 2018 Dec 21;6:369. doi: 10.3389/fpubh.2018.00369 (PMC6308159; doi:10.3389/fpubh.2018.00369)
Supplement: Supplementary file 1 [file Table_1.pdf]

Supplemental Table 1.

Table 1. Cell surface antibody used in the current study.

Epitope	Vendor	Cat. #	Lot	Fluorophore
CD34	Miltenyi Biotec	130095393	5151029045	VioBlue
CD31	BD Biosciences	555446	28577	PE
CD309	Miltenyi Biotec	130105303	5170629765	FITC
CD133	Miltenyi Biotec	120000426	5020703017	PE
CD45	BD Biosciences	555485	4184772	APC
CD73	BD Biosciences	561254	2237759	FITC
CD146	Miltenyi Biotec	130097942	5140217223	APC
