# Supplementary material for: Initial Assessment of Variability of Responses to Toxicants in Donor-Specific Endothelial Colony Forming Cells
Source: Front Public Health. 2018 Dec 21;6:369. doi: 10.3389/fpubh.2018.00369 (PMC6308159; doi:10.3389/fpubh.2018.00369)
Supplement: Supplementary file 2 [file Table_2.pdf]

Supplemental Table 2.

Table 2. Chemicals used in the current study.

Name	Chemical Formula	CAS #*	Purity	Catalog #	Range (μM)
Cadmium Chloride Hydrate	$CdCl_2 \cdot H_2O$	654054-66-7	99.995%	#529575	0.625-160
Zinc Chloride	$ZnCl_2$	7646-85-7	98%	#Z0152	1.560-400
Menadione	$C_6H_4(CO)_2C_2H$	58-27-5	98%	#M5625	0.625-160
Phytonadione	$C_{31}H_{46}O_2$	84-80-0	99.2%	# LRAA5981	0.390-100
Sodium (meta)arsenite	$NaAsO_2$	7784-46-5	≥90%	#S7400	0.390-100
Sodium arsenate dibasic heptahydrate	$Na_2HAsO_4 \cdot 7H_2O$	10048-95-0	98%	#A6756	0.390-100
Tributyltin Chloride	$C_{12}H_{27}S_nCl$	1461-22-9	96%	# T50202	0.078-20
Tin Chloride dihydrate	$SnCl_2 \cdot 2H_2O$	10025-69-1	98%	#243523	0.780-200

*CAS # – Chemical Abstracts Service number
