# Supplementary material for: Initial Assessment of Variability of Responses to Toxicants in Donor-Specific Endothelial Colony Forming Cells
Source: Front Public Health. 2018 Dec 21;6:369. doi: 10.3389/fpubh.2018.00369 (PMC6308159; doi:10.3389/fpubh.2018.00369)
Supplement: Supplementary file 4 [file Image_1.pdf]

Supplemental Figure 1. 384-well Plate Outline.

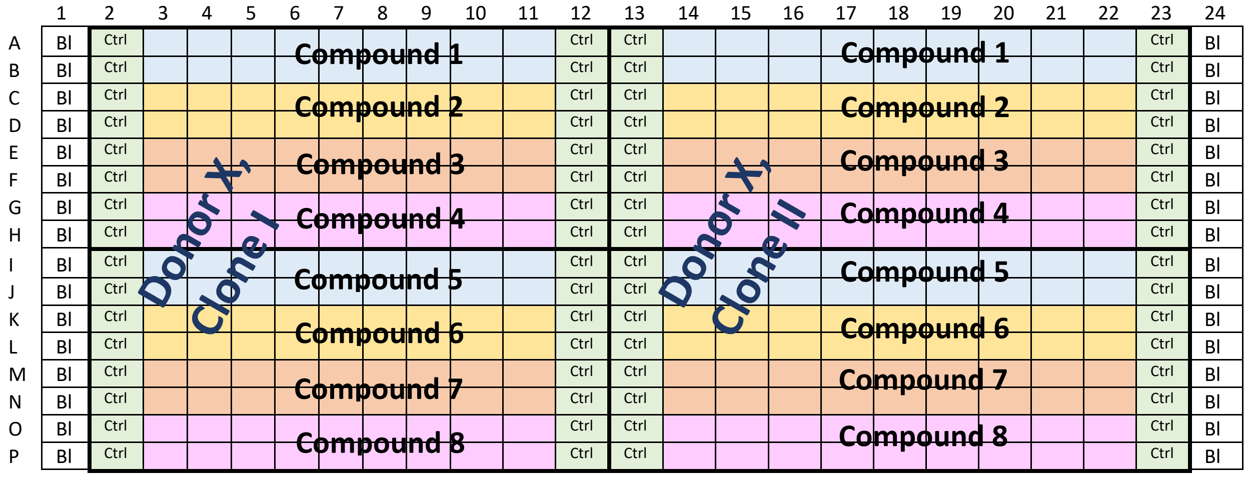

Bl – Blank (VecStem^{TM} cell growth media only)
Ctrl – Control (no treatment)

8 compounds were tested in 8 ECFC lines from 4 donors. Positioning of 2 ECFC clones per plate with 8 compounds/plate/clone. See also raw data in Supplemental Table 1 (Excel file).
