# Supplementary figures and images for: Initial Assessment of Variability of Responses to Toxicants in Donor-Specific Endothelial Colony Forming Cells
Source: Front Public Health. 2018 Dec 21;6:369. doi: 10.3389/fpubh.2018.00369 (PMC6308159; doi:10.3389/fpubh.2018.00369)

Supplemental Figure 2. Initial assessment of cytotoxicity in polyclonal CB002

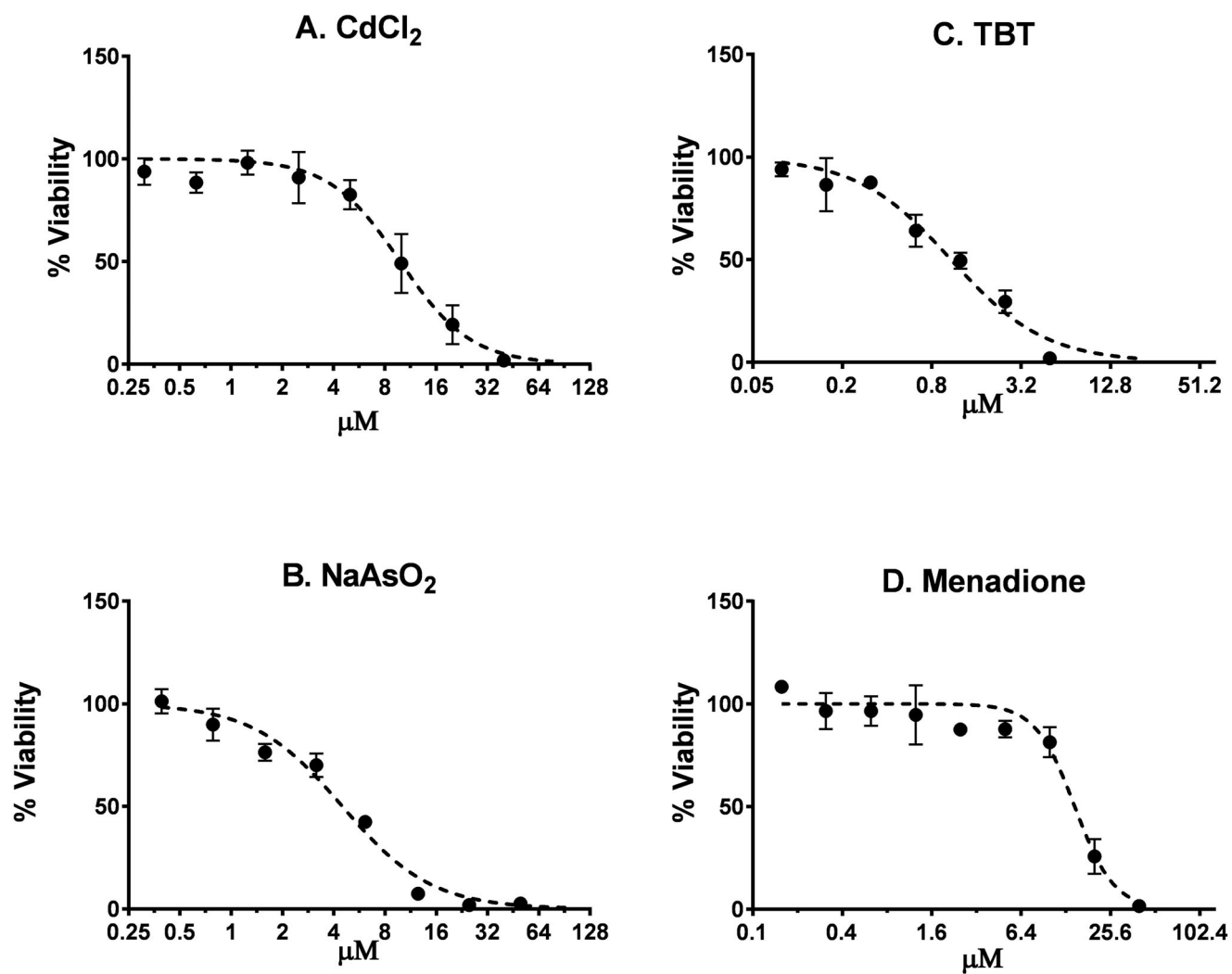

Supplement: Supplementary file 5 [file Image_2.pdf]
