# Supplementary material for: Initial Assessment of Variability of Responses to Toxicants in Donor-Specific Endothelial Colony Forming Cells
Source: Front Public Health. 2018 Dec 21;6:369. doi: 10.3389/fpubh.2018.00369 (PMC6308159; doi:10.3389/fpubh.2018.00369)

Supplemental Figure 3. Day-to-day variability of cytotoxic ECFCs responses to the selected set of toxicants. The X-axis shows concentration of toxicants on log scale (as indicated in Methods). The Y-axis shows estimated coefficient function from the model for the comparison of day 2 vs. day 1 (A) and day 3 vs. day 1 (B). Dotted lines represent point-wise lower and upper limits for 95% confidence interval for coefficient functions.

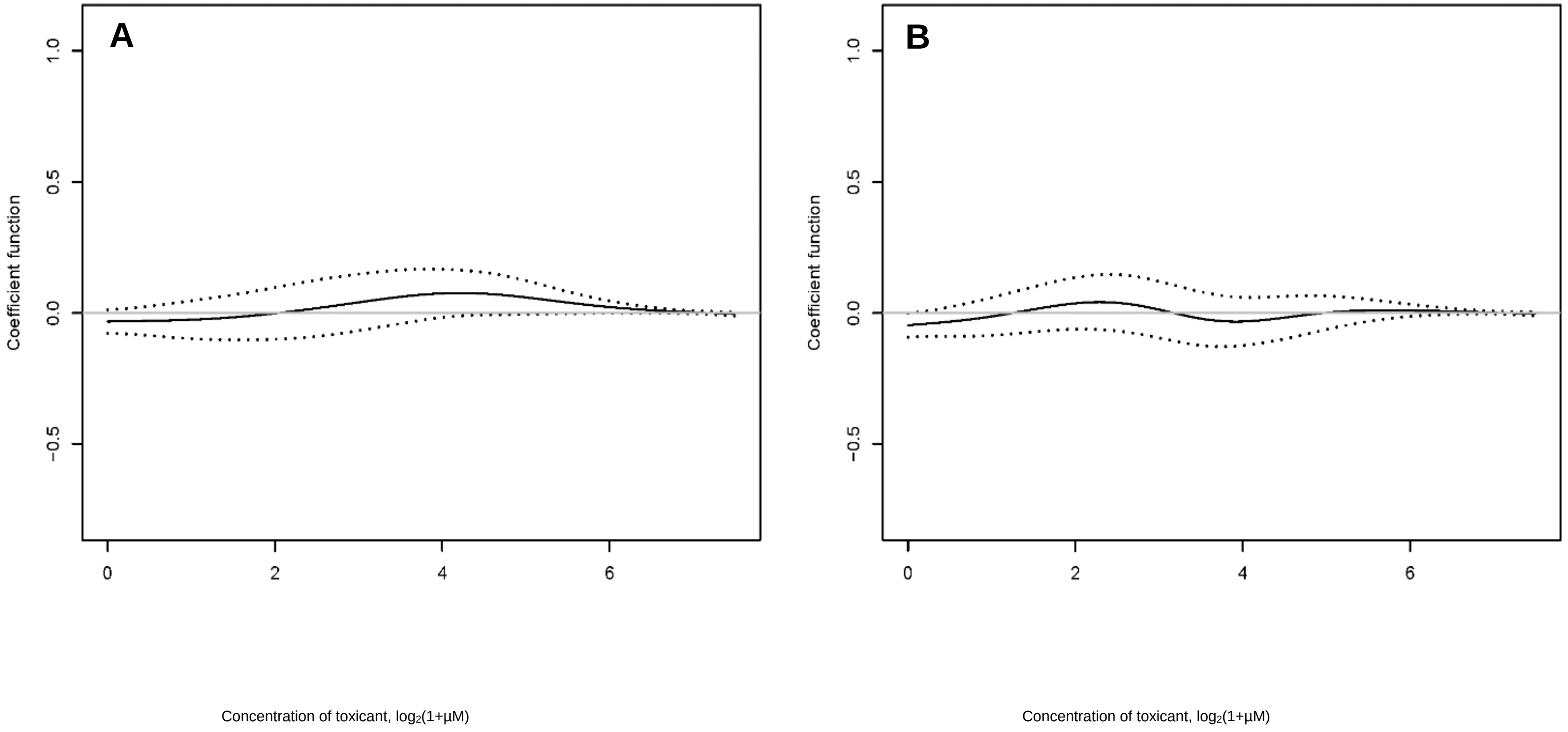

Supplement: Supplementary file 6 [file Image_3.pdf]
